# Hypersensitivity reaction after cyanoacrylate-adhesive closure of great saphenous vein requiring glue granuloma excision

**DOI:** 10.1016/j.jvscit.2026.102408

**Published:** 2026-07-14

**Authors:** Gillian J. Lee, Julianne Stoughton

**Affiliations:** Division of Vascular and Endovascular Surgery, Department of Surgery, Massachusetts General Hospital/Harvard School of Medicine, Boston, MA

**Keywords:** Cyanoacrylate, Endovenous glue, Granuloma, Hypersensitivity reaction, Non-thermal venous ablation, Venous insufficiency

## Abstract

Hypersensitivity reactions after cyanoacrylate-adhesive closure of incompetent saphenous veins can range from mild and self-limiting to more severe. In the mild form, they can present as phlebitis-like reactions that are transient and resolve with antihistamines and oral corticosteroids. Although severe reactions are still rare, they have been described several times in the literature before. We describe the case of a foreign body reaction after cyanoacrylate-adhesive closure of a great saphenous vein leading to glue extrusion requiring surgical excision. Consideration of individual patient and anatomic factors is important when deciding on the appropriate method for varicose vein ablation.

There are a wide range of treatment options for symptomatic varicose veins and axial incompetence. Cyanoacrylate (CA)-adhesive closure (CAC) using *n*-butyl-CA is one of several nonthermal nontumescent treatment methods available for endovenous ablation of incompetent saphenous veins.^1^ CAC has been demonstrated to be effective with a good safety profile and decreased rates of thermal injury.^2^ Potential complications include hypersensitivity reactions (HSRs). We report the case of a mild delayed HSR followed by a foreign body reaction and extrusion requiring excision. The patient provided consent for publication of case details and images.

## Case presentation

A 54-year-old woman presented to an outpatient vein clinic with symptoms of soreness, heaviness, itching, and fatigue of the left leg with swelling around the left ankle. Her past medical history was significant for prediabetes and rosacea. She had a history of skin irritation with Latex exposure, but no adverse reactions to adhesives or other allergies. She had previously undergone radiofrequency ablation of the right (contralateral) great saphenous vein (GSV) and polidocanol injectable foam of the below knee remnant. Duplex ultrasound examination demonstrated an incompetent 6.6 mm left GSV that came out of fascia in the mid-thigh and associated with large incompetent tributaries. Owing to the patient’s anatomy and presentation, endovenous CAC (VenaSeal Closure System, MedTronic) and ambulatory phlebectomy was planned for her Clinical, Etiological, Anatomical, and Pathophysiological class C3 chronic venous disease.

A 7F sheath was used to access the GSV below the knee and positioned 5 cm below the saphenofemoral junction. The CA was delivered through a 5F delivery catheter following the steps from the instructions for use with a total of 2.0 mL of CA used to treat a length of 53 cm. She simultaneously underwent phlebectomy of symptomatic left leg tributary veins with a total of 21 microincisions. There were eight microincisions made in the distal medial thigh over the most prominent varicose veins that were visible and palpable (Fig 1, *A*) along the length of the incompetent GSV.

**Fig 1:**
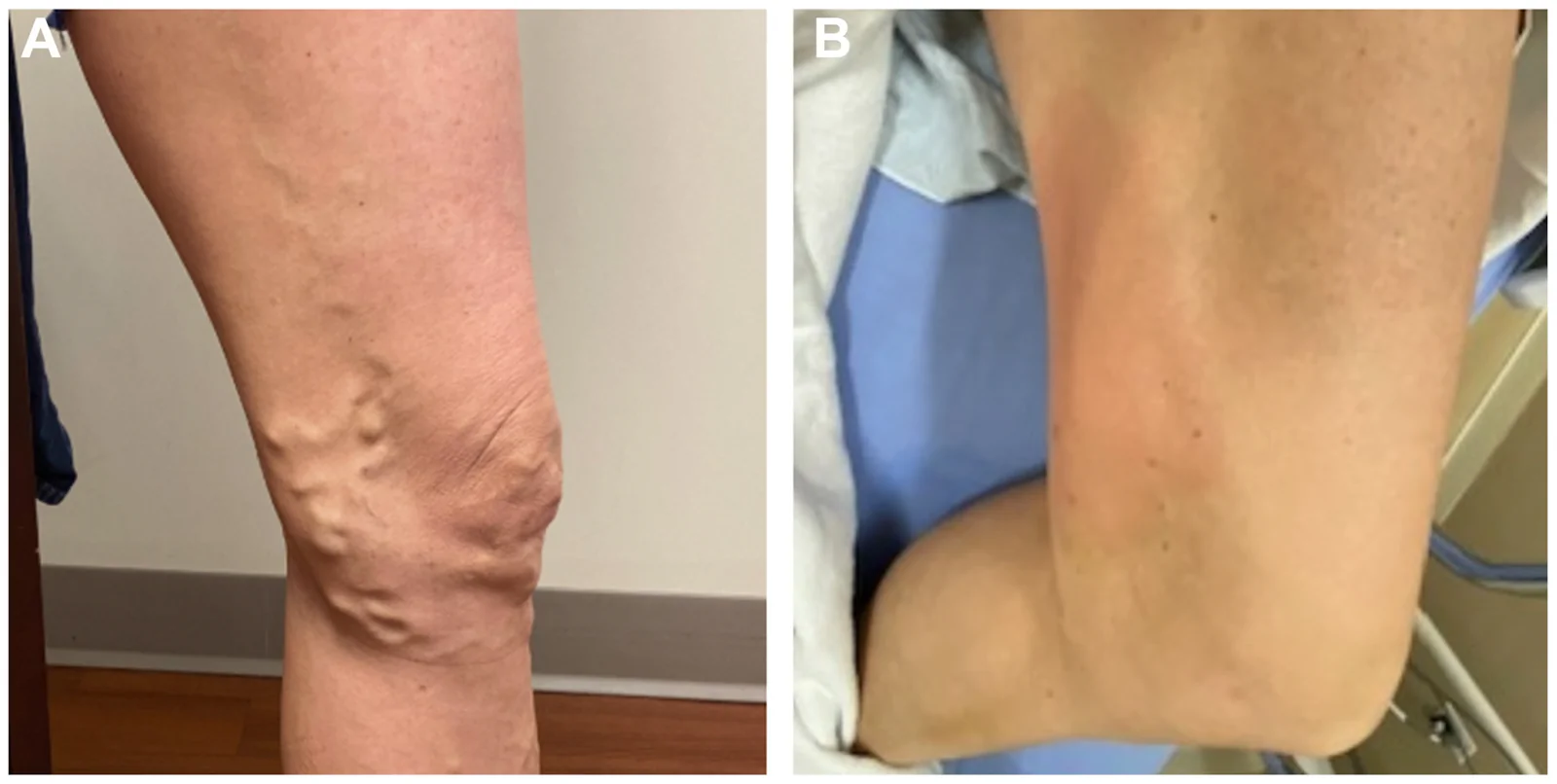
**A,** Photograph showing left medial thigh before endovenous cyanoacrylate-adhesive closure and ambulatory phlebectomies. **B,** Photograph showing left medial thigh after the procedure with erythema but ambulatory phlebectomy incisions well-healed.

Twenty-seven days postprocedure, the patient developed erythema and mild swelling in the distal medial thigh overlying the superficial segment of the GSV without systemic symptoms including fevers and chills (Fig 1, *B*). She presented to an outside Emergency Department where she underwent a duplex ultrasound examination that did not demonstrate any deep vein thrombosis or superficial thrombophlebitis. She was prescribed a 10-day course of cephalexin for a presumed diagnosis of cellulitis. At the 1-month follow-up visit, her local hypersensitivity symptoms had resolved without steroid treatment. At the 4-month follow-up visit, she was noted to have a palpable, <1 cm, nontender nodule in the distal medial thigh but otherwise no other symptoms.

Two years later, the patient presented with new-onset small volume serous drainage from the medial thigh nodule. The nodule itself was firm and nontender with a small punctate eschar and 1 cm of surrounding erythema (Fig 2, *A*). Firm CA was palpable along the course of the GSV from the midthigh to the knee. A duplex ultrasound examination demonstrated a noncompressible heterogeneous lesion associated with the nodule (Fig 2, *B*).

**Fig 2:**
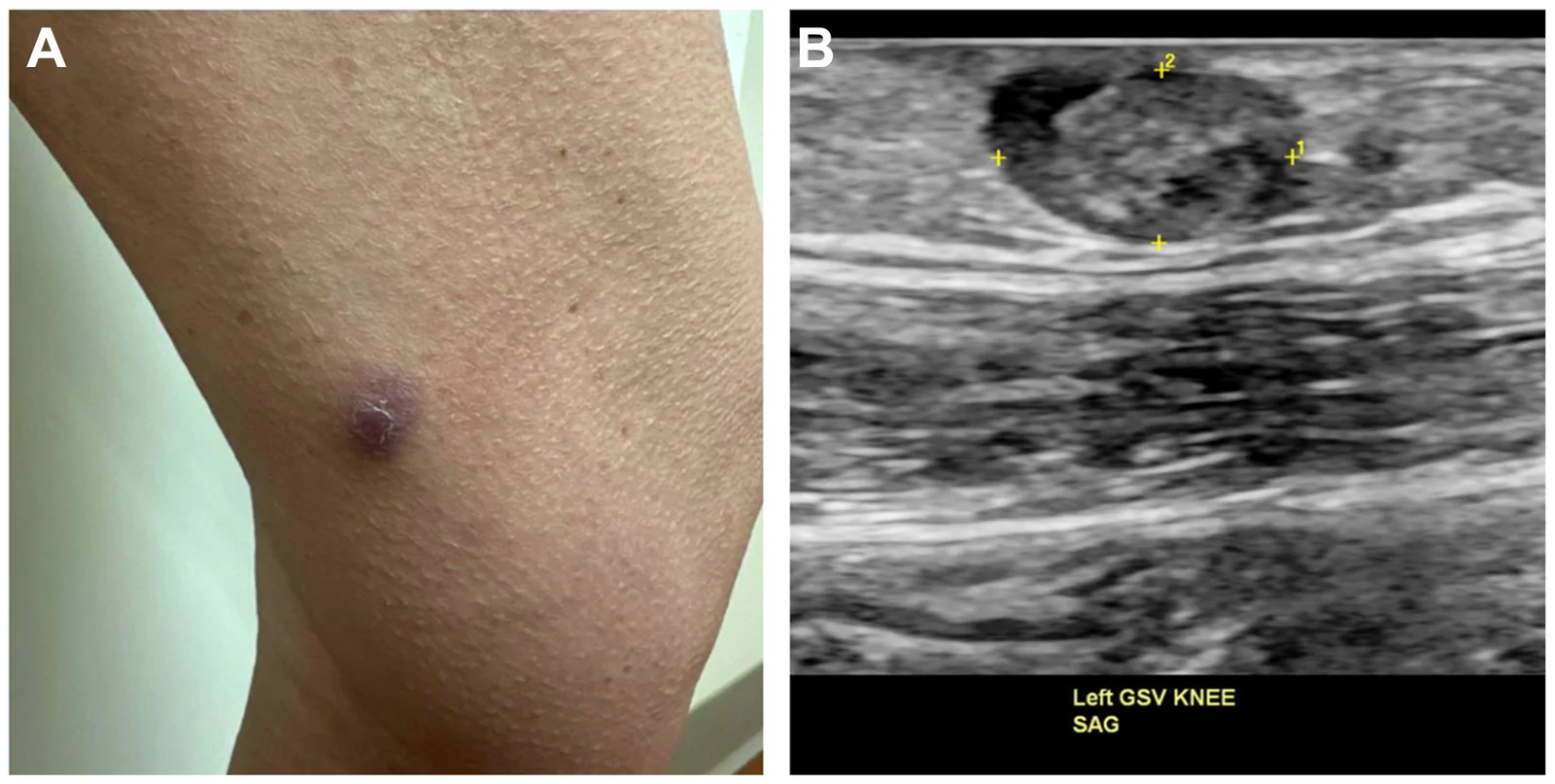
**A,** Pre-excision photograph of the nodule in the distal medial thigh with punctate eschar. **B,** Ultrasound image demonstrating a subcutaneous heterogenous lesion, underlying the nodule in the distal medial thigh.

Given the concern of foreign body reaction and CA extrusion, she underwent local excision of the nodule in the office. An elliptical incision was made surrounding the nodule (Fig 3, *A*) and was carried down to the subcutaneous tissue to excise the friable cyst (Fig 3, *B*). There were several small foreign bodies at the base of the cyst resembling CA that were removed (Fig 3, *C*). Pathology demonstrated granulation tissue with acute and chronic inflammation and foreign body giant cell reaction to foreign material (Fig 4, *A* and *B*). At up to 2 years after excision, the patient had complete resolution of symptoms with no further recurrence of HSRs, and the wound remains well healed (Fig 5).

**Fig 3:**
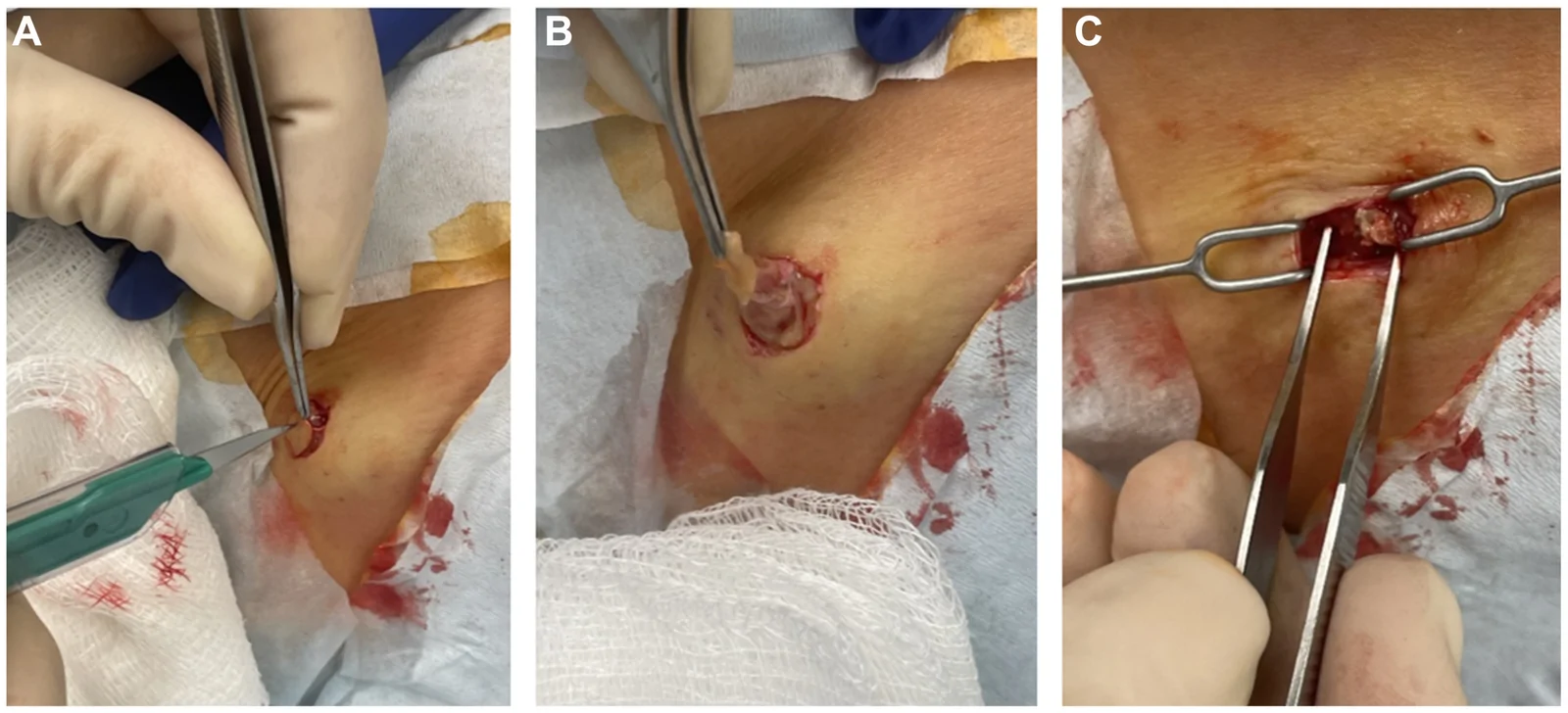
Local excision of granulomatous tissue. **A,** Elliptical incision made. **B,** Dissection and excision of granuloma. **C,** Small foreign bodies removed from base of granuloma.

**Fig 4:**
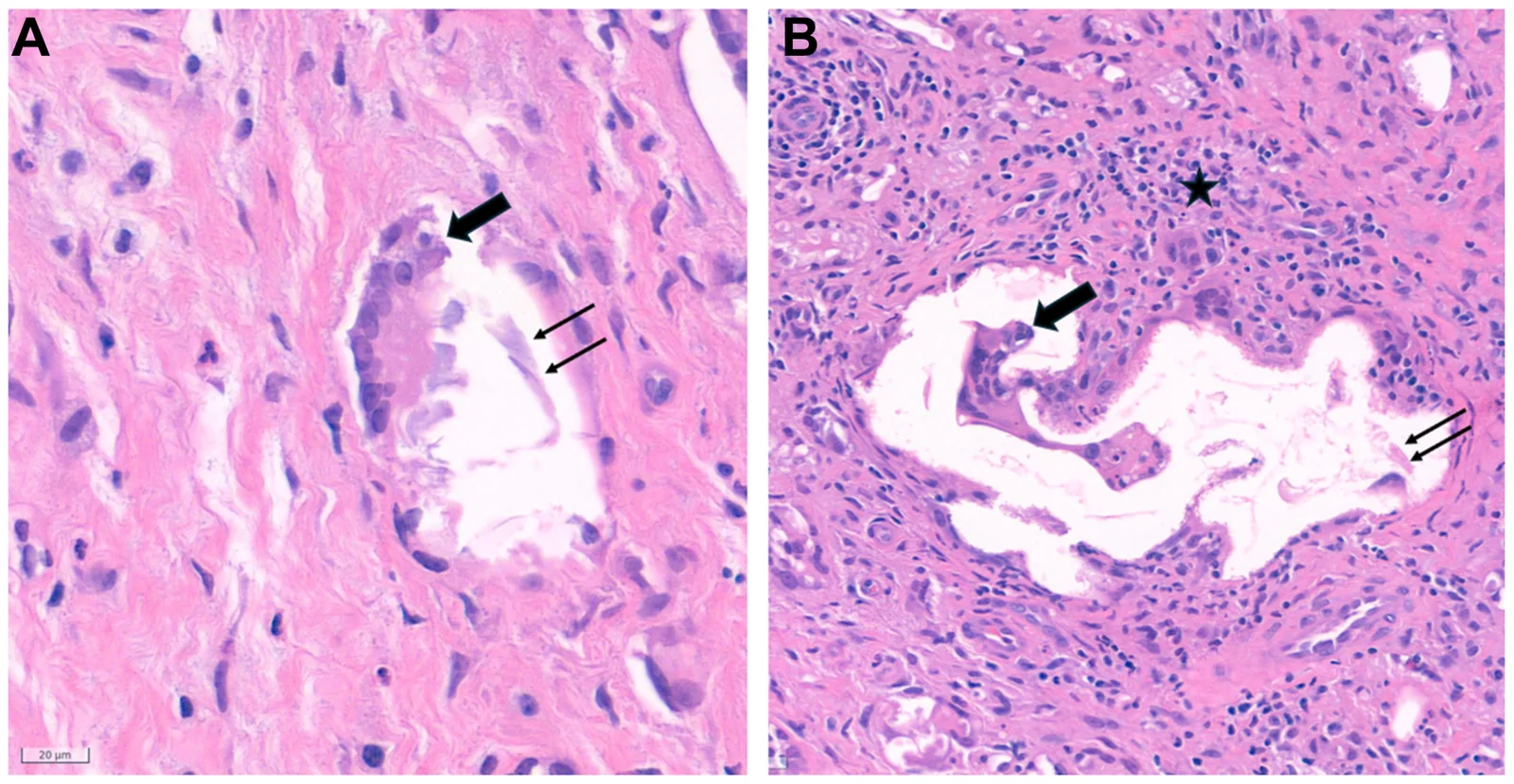
Histopathologic evaluation of the excised tissue with multinucleated foreign body giant cells (*thick arrows*), foreign material (*thin arrows*), and histiolymphocytic inflammation (*star*). **A,** Hematoxylin and eosin stain; ×40 magnification. **B,** Hematoxylin and eosin stain; ×20 magnification.

**Fig 5:**
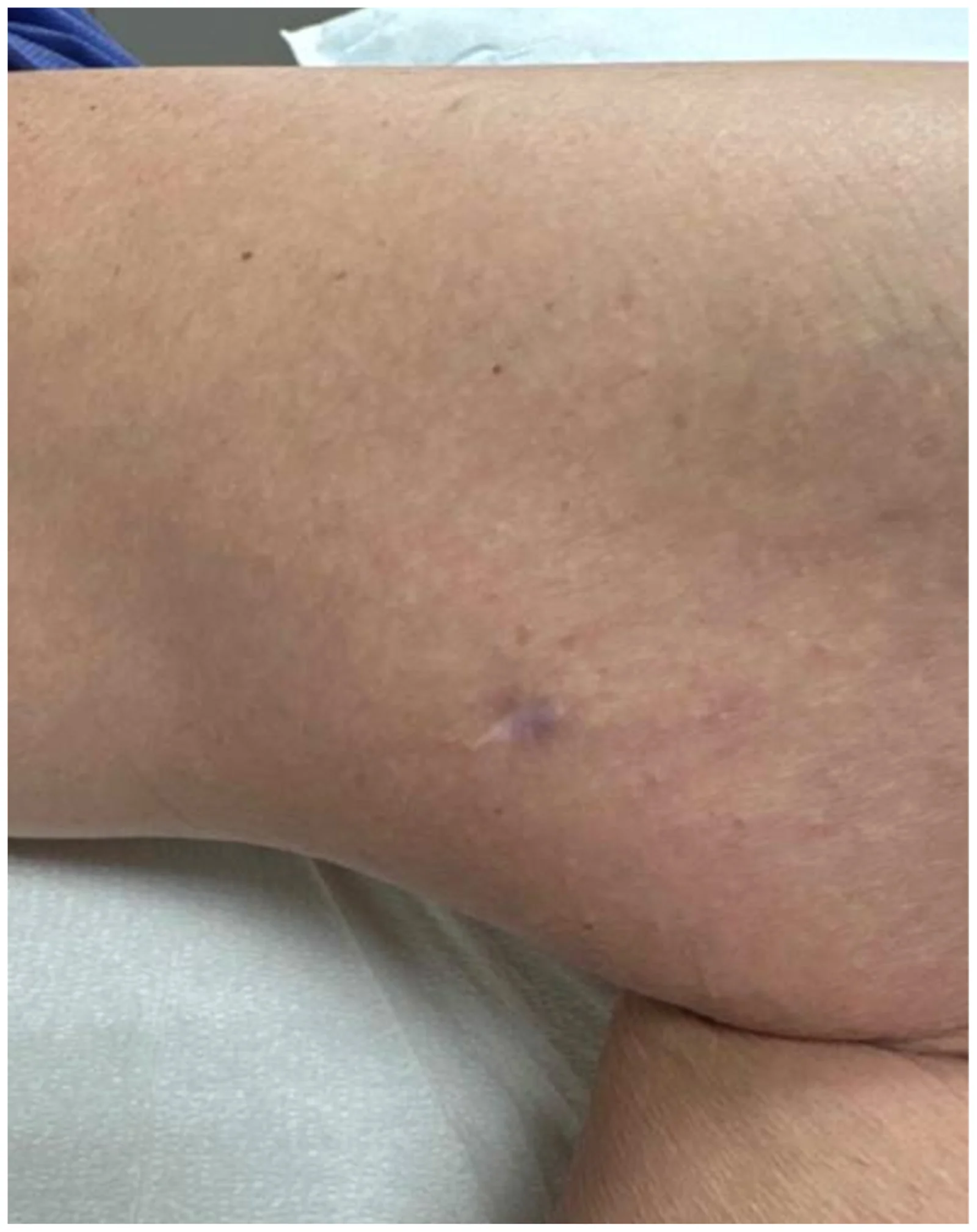
Photograph showing left medial thigh 2 years after granuloma excision procedure.

## Discussion

Recent studies have demonstrated the incidence of HSRs after CAC to range from 6.0% to 21.2%.^3^, ^4^, ^5^ Symptoms can include a phlebitis-like reaction with erythema, itching, swelling, and pain/tenderness over the treated vein that develops approximately 1 week after the procedure.^6^^,^^7^ This reaction has been differentiated from the typical phlebitis that occurs after endothermal ablations^8^ and is postulated to be a type IV HSR. Management regimens have included nonsteroidal anti-inflammatory drugs, antihistamines, and topical and oral corticosteroids for more severe cases.^6^^,^^7^^,^^9^ Most HSRs are mild and self-limited after medical treatment.

Although *n*-butyl CA patch allergy testing is possible, it is not a commercially available patch testing allergen and may not always be predictive of these HSRs.^10^ Retrospective single-institution studies have found other risk factors for developing HSRs to include: saphenous size of >8 mm and suprafascial saphenous veins <1 cm from the skin, as well as younger age, female sex, and history of allergies.^4^^,^^7^ Our patient had a sensitivity to Latex which may have increased her risk of developing the HSR 4 days after the CAC procedure which resolved quickly without corticosteroids or any further interventions.

There has been another reported case of glue extrusion in a patient with no previous allergic or HSRs who presented 9 months after CAC of the GSV with erythema over a nontender nodule over the medial thigh that ultimately required surgical excision. Histological analysis was consistent with a chronic inflammatory response demonstrating multinucleated giant cells surrounding glue.^11^ Other case reports have similarly demonstrated foreign body reactions to intraluminal foreign material.^9^^,^^12^ The findings from our patient add to the existing literature that the presence of foreign body cells after CAC is a physiological response in all patients who have had pathologic evaluation based on five separate studies up to 5 years after closure.^13^ Some patients are asymptomatic, while in more severe, surgical excision may be necessary.

In addition, our patient simultaneously underwent ambulatory phlebectomy of tributary veins. We hypothesize the glue may have initially extravasated into a small tributary vein that was then interrupted with phlebectomy leading to its extrusion. At present time, we recommend careful patient selection when considering CAC as a treatment modality such as in patients with venous leg ulcers that require ablation of the entire length of GSV and tributaries without risk of thermal injury, or in those with mixed arterial and venous disease who are unable to tolerate substantial compression. Otherwise, CAC should be used only after consideration of patient-specific factors including allergies and anatomy, and after a thorough risk benefit discussion with the patient including counseling on the risk of adverse events that are either self-limiting or ultimately require surgical interventions. Ambulatory phlebectomy should be performed in a staged fashion if required, and care should be given to avoid subdermal CA exposure when removing the catheter from the vein to reduce the risk of inflammation or granuloma formation at the exit site.

## Conclusions

HSRs to CA are not uncommon, and its use for varicose vein ablation requires consideration of patient factors and other concurrent procedures that are planned. HSRs are typically successfully treated with medical management. Foreign body reactions occur in all patients who undergo CAC but can result in rare but more serious complications that require surgical excision depending on patient presentation.

## Funding

There were no sources of funding for this study.

## Disclosures

The authors have no conflicts of interest to disclose.
